# Moving Beyond Fenton-Based Peroxidase Nanozymes for Analytical Sensing: Perspectives on Nonradical Alternatives from Heterogeneous Catalysis

**DOI:** 10.1021/acs.analchem.6c01457

**Published:** 2026-06-01

**Authors:** Bo Yuan, Yifan Cui, Pi-Tai Chou, Yung-Kang Peng

**Affiliations:** † Department of Chemistry, 53025 City University of Hong Kong, Kowloon, Hong Kong SAR 999077, China; ‡ Department of Chemistry, 33561 National Taiwan University, Taipei 106319, Taiwan

## Abstract

Artificial enzymes have garnered attention for their potential to overcome the cost and stability limitations of natural enzymes. Among these, nanozymes—solid nanomaterials with enzymatic activity—have emerged as promising alternatives due to their facile synthesis and recyclability. To date, over half of the reported nanozymes mimic peroxidase (POD), catalyzing the oxidation of chromogenic substrates like 3,3′,5,5′-tetramethylbenzidine (TMB) with H_2_O_2_. While many studies have successfully demonstrated the substitution of horseradish peroxidase (HRP) with POD nanozymes in various sensing applications, challenges persist in characterizing their active sites to establish reliable catalytic correlations. Furthermore, unlike HRP, which utilizes H_2_O_2_ stoichiometrically for TMB oxidation, most POD nanozymes activate H_2_O_2_ through radical pathways. This results in low H_2_O_2_ utilization, hindering their practical use in quantitative assays compared to HRP. This perspective offers potential solutions from the standpoint of heterogeneous catalysis, including the use of probe-assisted surface techniques to distinguish active-site reactivity and the design of POD nanozymes that activate H_2_O_2_ via nonradical pathways to improve H_2_O_2_-to-substrate stoichiometry. These insights are expected to guide the future development of POD nanozymes, shifting the focus from maximizing OH radical generation to achieving controlled nonradical H_2_O_2_ activation and thus better mimicking HRP’s catalytic behavior.

## Graphical abstract

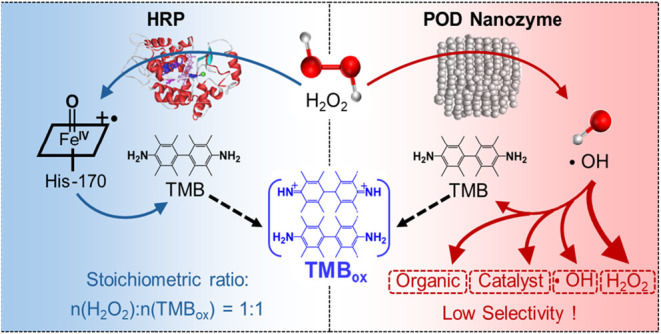

## Introduction

The design and synthesis of artificial enzymes have attracted significant attention over the past few decades due to their potential for lower costs and enhanced thermal/pH stability compared to natural enzymes.^1,2^ Among these, solid nanomaterials that catalyze enzymatic reactions—known as nanozymes—have emerged as a promising alternative.^3–6^ By definition, nanozymes are heterogeneous catalysts that exist in a different phase from the molecular reactants, thereby offering additional advantages for practical applications, such as ease of preparation, separation, and recyclability. This field has rapidly expanded in recent years, as evidenced by a significant increase in publications and citations (Scheme 1a) along with a wide range of applications across diverse areas (Scheme 1b), including healthcare and therapeutic interventions,^7,8^ biomolecular and pollutant sensing,^9,10^ and antibacterial and antifouling applications.^11^ Its significance to the scientific community was highlighted by International Union of Pure and Applied Chemistry (IUPAC) as one of the top 10 topics of 2022 and as one of the top 10 emerging technologies of 2025 by the World Economic Forum.

**Scheme 1:**
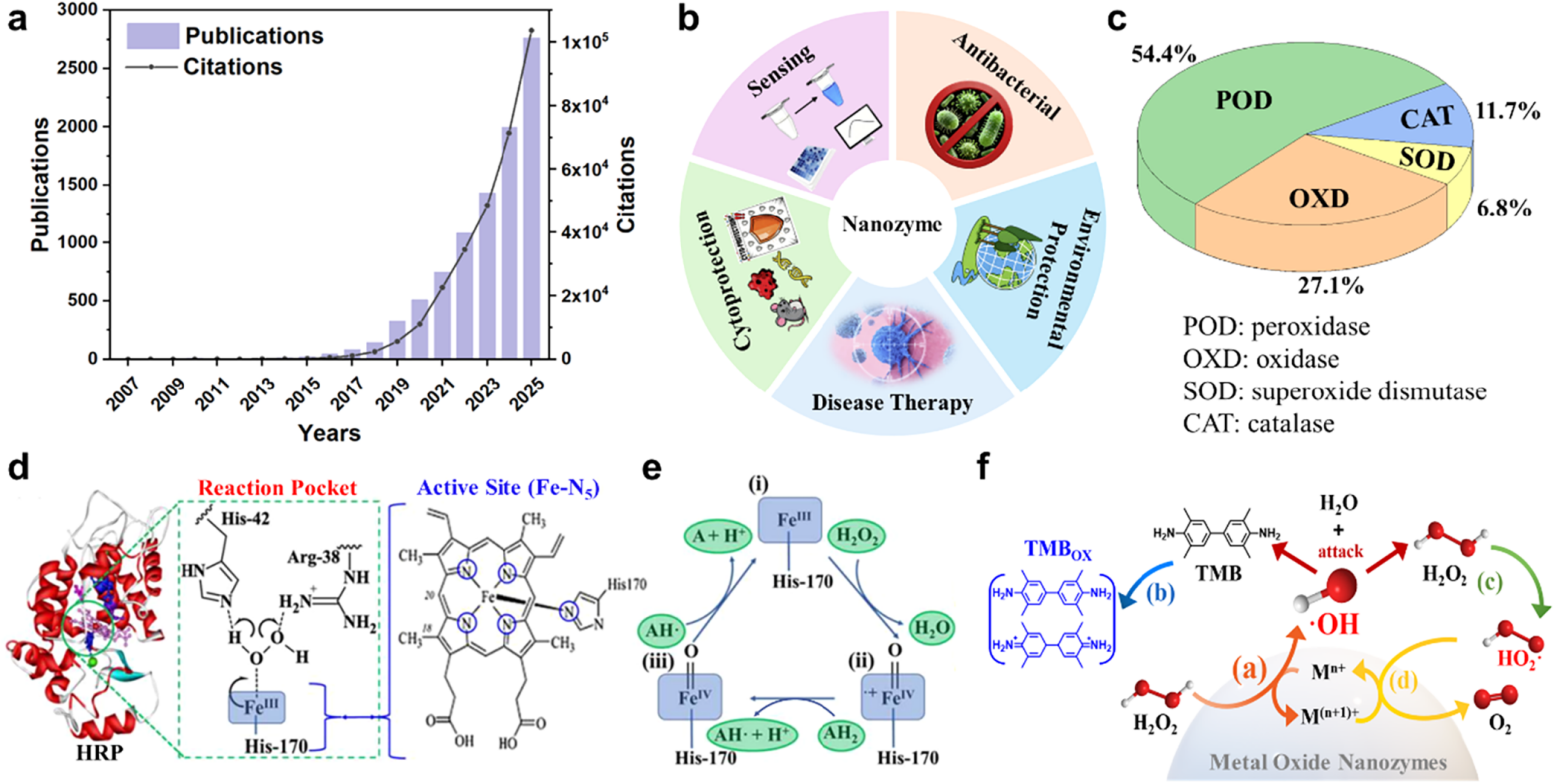
(a) Publication and Citation Trends in the Field of Nanozymes since 2007 (Data from Web of Science Using the Key Word “Nanozymes”), (b) Diverse Applications of Nanozymes across Various Fields, (c) Distribution of Nanozymes Mimicking Different Types of Oxidoreductases, (d) The Reaction Pocket of HRP and (e) Its Corresponding Catalytic Cycle, and (f) The Catalytic Cycle Proposed for Fenton-Based POD Nanozymes^a^

a Reproduced with permission from ref 25. Copyright 2022 American Chemical Society

Various nanozymes have been developed to mimic a wide range of natural enzymes such as peroxidase,^12^ oxidase,^13,14^ superoxide dismutase,^15^ catalase,^16^ carbonic anhydrase,^17^ and hydrolase.^18^ Despite this diversity, over 96% of the reported nanozymes primarily exhibit oxidoreductase-like activity, with more than half specifically mimicking peroxidase (POD) (Scheme 1c). Horseradish peroxidase (HRP), the most representative natural enzyme of this kind, is widely used todays, with chromogenic substrates such as 3,3′,5,5′-tetramethylbenzidine (TMB) commonly found in various assay kits, immunohistochemistry for Western blotting, and enzyme-linked immunosorbent assays (ELISAs).^19^ Mechanistic studies of HRP reveal that the reaction pocket consists of an Fe­(III)-protoporphyrin IX and His-170 as the proximal ligand (Scheme 1d).^20^ Two amino acids, His-42 and Arg-38, located above are crucial for orienting H_2_O_2_ and facilitating O–O bond activation. During the reaction (Scheme 1e), H_2_O_2_ first oxidizes Fe­(III) (compound (i)) to Fe­(IV)-oxo with a retained π-cation radical on the porphyrin ring (compound (ii)). This reactive species then oxidizes TMB to regenerate Fe­(III) via Fe­(IV)=O (compound (iii)), completing the catalytic cycle. This property enables HRP to be used in various sensing applications to stoichiometrically catalyze the oxidation of TMB with H_2_O_2_, resulting in a measurable color change from colorless to bluish TMB_ox_ at 652 nm for analyte quantification.

The discovery of POD nanozymes dates back to year 2007, when Gao et al. found that Fe_3_O_4_ nanoparticles (NPs) can oxidize TMB with H_2_O_2_ due to switchable oxidation states.^21^ Since then, many other transition metal oxides, as well as metals and their alloys, have been reported to exhibit POD-like activity.^3–6^ The use of TMB has also become essential in subsequent studies for reporting and comparing material activity, as standardized in protocols within the nanozyme community.^22,23^ While the key species oxidizing TMB is under debate, a general consensus holds that OH radicals, rather than surface high-valent species (e.g., Fe­(IV)=O), are the dominant oxidant.^24^ This is understandable because AH_2_ oxidation by HRP is initiated by Fe­(IV)=O with a retained radical (compound (ii)) rather than the less reactive Fe­(IV)=O (compound (iii)) (Scheme 1e). Based on Fenton catalysis, Yuan et al. revealed the full catalytic cycle for these POD nanozymes, involving both OH and HO_2_ radicals.^25^ As shown in Scheme 1f, H_2_O_2_ is initially reduced by M^n+^ in these POD nanozymes to generate free OH radicals (step (a)), which subsequently produce TMB_ox_ (step (b)) for color change and HO_2_ radicals (step (c)) for M^n+^ regeneration (step (d)). Although this Fenton-based mechanism has successfully guided the design of various highly active POD nanozymes, a key challenge remains in establishing reliable catalytic correlations, particularly in resolving the chemical state (or reactivity) of active species. Additionally, the POD-like activity of these nanozymes is inherently limited by numerous Fenton/Fenton-like reactions that consume OH/HO_2_ radicals beyond those listed in Scheme 1f, which affects their H_2_O_2_ utilization and sensing performance.

Considering that researchers in the field of nanozymes may not be familiar with catalysis, this perspective aims to provide insights from heterogeneous catalysis and seeks to address these challenges. We will first discuss the limitations of X-ray photoelectron spectroscopy (XPS), commonly used in the field for catalytic correlations, and encourage the use of surface probe techniques that have been routinely employed in heterogeneous catalysis for this purpose. Then, we will reveal that OH radicals, due to their strong oxidation potential and nonselective nature, can be easily scavenged by various substances in the stock solution, including the nanozymes themselves. Their performance in biomolecule and pollutant sensing may thus be further diminished by the presence of various species in complex biological and environmental matrices. Drawing inspiration from catalysts that utilize H_2_O_2_ for selective oxidation in heterogeneous catalysis, the design of POD nanozymes that activate H_2_O_2_ via a peroxo/oxo pathway, akin to that of HRP, is believed to improve substrate selectivity and hence facilitate stoichiometric H_2_O_2_ utilization in the relevant sensing applications.

## Fenton-Based Peroxidase Nanozymes

In this section, we discuss the key factor determining the observed activity of Fenton-based POD nanozymes across different materials. The correlation will be further verified in pristine materials that exhibit shape-dependent and single-atom/cluster-dependent POD-like activity.

### Fenton Catalysis and POD Nanozymes

The Fenton reaction, discovered by Henry John Horstman Fenton in the 1890s, generates OH radicals (+2.8 V_NHE_) by reacting H_2_O_2_ with Fe^2+^ and has gained prominence for degrading organic pollutants in wastewater treatment.^26^ Over the years, researchers have built on this foundational work to explore other transition metal-based heterogeneous catalysts for greater efficiency and versatility. Nowadays, these catalysts have found applications in various fields, especially in advanced oxidation processes (AOP) for environmental remediation^27^ and nanomedicine for extensive biomedical applications.^28^ The development of POD nanozymes, although relatively late compared to these fields, is believed to also adhere to the principles aimed at enhancing the conversion of H_2_O_2_ to OH radicals (i.e., step (a) in Scheme 1f), thereby increasing TMB oxidation and POD-like activity. Since step (a) is the rate-limiting step in the catalytic cycle,^25^ the electron richness of a given active metal “M” should thus positively contribute to the activity of a POD nanozyme. To provide an overview of POD-like activity from diverse material classes, Cai et al. normalized their observed activities by surface area across noble metals, alloys, and metal oxides (Figure 1a).^29^ As expected, noble metals (e.g., Pt, Pd, Ru, Ir) and their alloys generally exhibit higher POD-like activity than that of 3d transition metal oxides, due to the greater electron richness of metallic M relative to its cationic oxide forms. Although less active, 3d transition metal oxides are preferred for practical applications due to their natural abundance and cost-effectiveness. Given this, Yuan et al. further explored the catalytic correlation between the oxidation states of these metal cations and their corresponding POD-like activity.^25^ Taking Cu/Fe-based oxides as an example, their activity order (Cu_2_O > CuO > Fe_3_O_4_ > Fe_2_O_3_) matches the trend in Figure 1a and positively correlates with the reaction rate of the corresponding cations toward H_2_O_2_ (Cu­(I) > Cu­(II) > Fe­(II) > Fe­(III), Figure 1b). This may explain why researchers have recently shifted their focus from CuO^30,31^ to Cu_2_O and Cu­(I)-based materials^29,32,33^ as POD nanozymes. Similarly, Fe_2_O_3_ has been less studied than Fe_3_O_4_ as a POD nanozyme in the literature.^3–6^ Further analysis of other 3d transition metal oxides led the authors to conclude that, for a given active metal “M”, a lower oxidation state (or higher in electron density) results in a faster reduction of H_2_O_2_ to OH radicals and enhanced TMB oxidation (Figure 1c­(i)). A similar catalytic correlation can also be observed in Fenton catalysts used in other fields mentioned above.^27,28^

**Figure 1:**
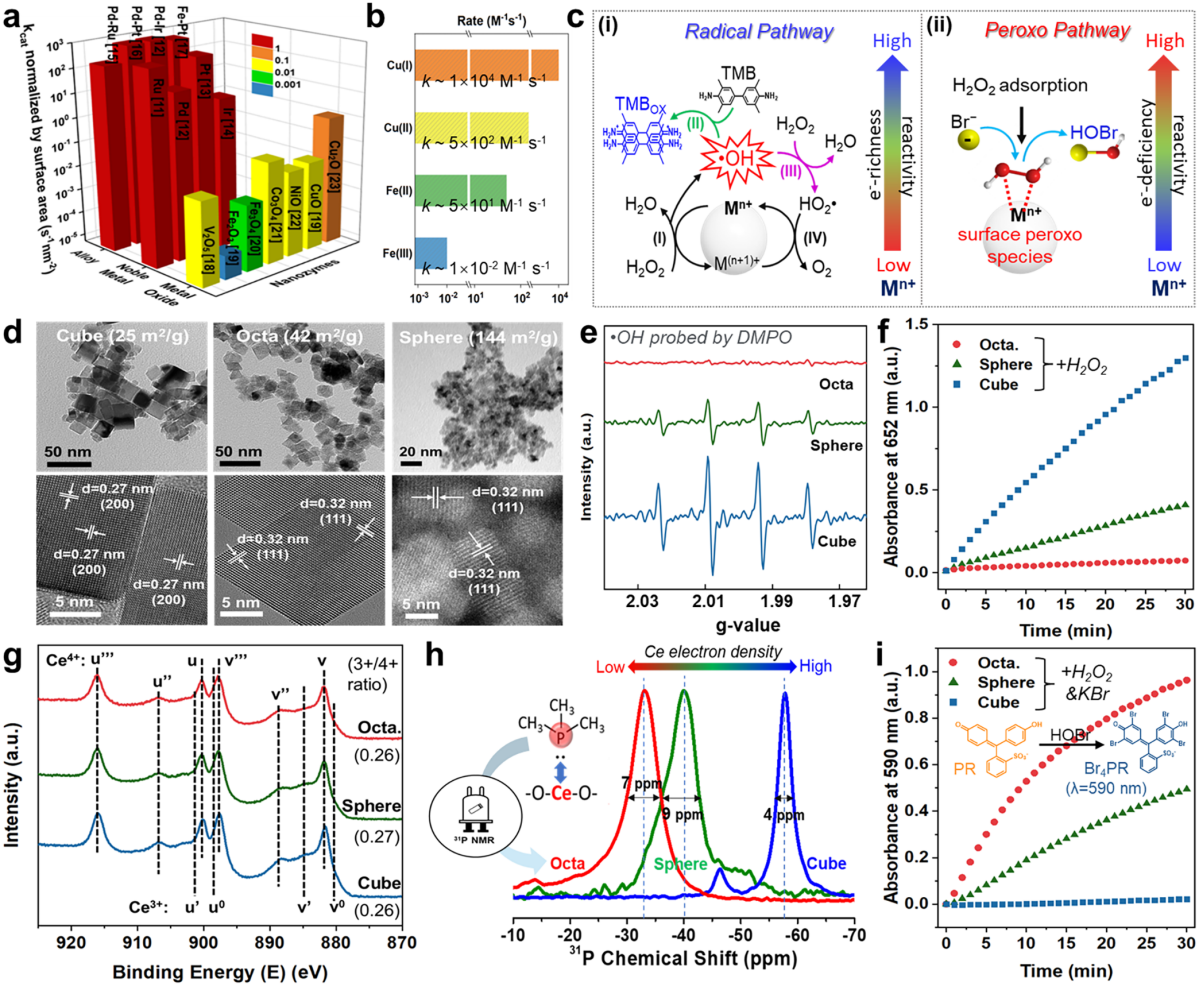
(a) Normalized POD-like activity of noble metals, alloys, and metal oxides reported in the literature. Reproduced with permission from ref 29. Copyright 2025 American Chemical Society. (b) Reported reaction rates of Cu­(I), Cu­(II), Fe­(II), and Fe­(III) with H_2_O_2_. Reproduced with permission from ref 25. Copyright 2022 American Chemical Society. (c) H_2_O_2_ activation via (i) the radical pathway for POD mimicking and (ii) the peroxo pathway for bromoperoxidase (BPO) mimicking. (d) Transmission electron microscope (TEM) images and surface area of CeO_2_ cube, octa, and sphere. Their (e) production of OH radicals probed by DMPO-assisted EPR, (f) POD-like activity, and (g) Ce_3d_ XPS spectra. Figures (d–g) reproduced with permission from ref 39. Copyright 2023 American Chemical Society. (h) TMP-assisted ^31^P NMR spectra of CeO_2_ shapes. Reproduced with permission from ref 41. Copyright 2020 American Chemical Society. (i) BPO-like activity of CeO_2_ shapes. Reproduced with permission from ref 39. Copyright 2023 American Chemical Society.

Table 1 compares several Fenton-based POD nanozymes discussed in this perspective with respect to their key active species, H_2_O_2_ activation pathway, and activity. Although Cu­(II) and Fe­(III) are less capable than their lower-oxidation-state counterparts of reducing H_2_O_2_ to OH radicals, the adsorbed H_2_O_2_ is instead expected to be activated as peroxo/oxo species on them. In addition to the effect of oxidation states on POD-like activity, we will further explore other factors in this section, including the exposed surfaces of CeO_2_, the electronic structures of Ru in both single-atom and cluster forms, and the location effects of Fe–N_4_ single sites within the support material.

**Table 1:** Comparison of POD Nanozymes Based on Their Key Surface Species, H_2_O_2_ Activation Pathway, and Activity

			H_2_O_2_ activation pathway			
material	systems	key surface species	radical	peroxo/oxo	POD-like activity	refs.
Cu-based oxides	CuO	Cu(II)		dominant	lower	^25^
	Cu_2_O	Cu(I)	dominant		higher	
Fe-based oxides	Fe_2_O_3_	Fe(III)		dominant	lower	^25^
	Fe_3_O_4_	Fe(II)	dominant		higher	
CeO_2_	(111) surface	electron-lean Ce		∼100%	negligible	^39^
	(100) surface	electron-rich Ce	∼100%		high	
Ru supported on MgO	single-atom	positively charged Ru		dominant	lower	^62^
	cluster	metallic Ru	dominant		higher	
Fe–N_4_ supported on carbon	on basal plan	electron-lean Fe		dominant	lower	^65^
	on edge	electron-rich Fe	dominant		higher	^66^

### Catalytic Correlation for CeO_2_ Shapes

To obtain POD nanozymes with improved activity, a wide range of synthetic approaches and surface modification strategies have been developed over the past few years.^3–6^ Researchers primarily use XPS to assess the oxidation states of the active metal “M” and their relative abundances, establishing correlations with the produced OH radicals and the observed POD-like activity. However, this technique has faced challenges as it collects photoelectrons not only from the sample’s topmost surface but also from subatomic layers that do not participate in the reaction. For instance, conventional XPS using Al as the source (1486.6 eV) excites Ce 3d photoelectrons with a kinetic energy of about 550 eV, providing information from within 5–7 nm of the surface.^34^ The oxidation state of Ce at the topmost surface is thus significantly diluted by Ce signals from the subsurface or bulk. Additionally, the “discrete” oxidation states provided by XPS (e.g., either 3+ or 4+ for Ce) do not reflect the electron density of surface Ce, which is believed to vary continuously with local coordination structures and atomic arrangements.^35,36^ This explains why pristine CeO_2_ NPs without a certain degree of surface modification often exhibit similar Ce_3d_ XPS spectra,^37,38^ making it challenging to establish a reliable correlation with the produced OH radicals and hence POD-like activity. For example, in our recent report,^39^ we prepared cubic and octahedral CeO_2_ NPs, enclosed by well-defined (100) and (111) surfaces (Figure 1d), respectively, along with spherical CeO_2_ NPs having rough surfaces. These CeO_2_ shapes exhibit distinct OH radical production (Figure 1e) and POD-like activities (Figure 1f). However, they display nearly identical XPS Ce_3d_ spectra and comparable Ce^3+^/Ce^4+^ ratios of approximately 0.26 (Figure 1g).^39^ While AP-XPS (atmospheric pressure or near ambient pressure) has proven valuable for exploring surface chemistry,^40^ the Ce^3+^/Ce^4+^ ratios obtained for these CeO_2_ shapes do not differ substantially from the conventional case due to inherent detection depth issue.^41^

To address this, researchers in heterogeneous catalysis employ probe-assisted techniques, such as ammonia-temperature programmed desorption (TPD),^42^ pyridine-infrared (IR),^43^ and nuclear magnetic resonance (NMR) with NMR-active probes,^44^ to assess and differentiate the electron density (or Lewis acidity) of surface metal cations. Among these methods, using ^31^P NMR along with the Lewis basic molecule, trimethylphosphine (TMP), as a surface probe^45–48^ has proven the most effective in differentiating the electron density of surface Ce sites across these CeO_2_ shapes.^41^ This is because the stronger Lewis acidity of the surface Ce sites forms more robust adduct bonds with TMP, resulting in a shift of δ^31^P toward positive values. Figure 1h shows that surface Ce sites on the (100) surface of cube exhibit the highest electron density, followed by the rough surface of sphere and the (111) surface of octahedron (denoted as octa).^41^ This trend aligns well with the observed OH radical production and the corresponding POD-like activity among CeO_2_ shapes, confirming again that the higher an electron density for a given active metal “M”, the faster it can produce OH radicals, leading to better POD-like activity (Figure 1c­(i)). Although the electron-lean Ce sites on the (111) surface of CeO_2_ octa cannot reduce H_2_O_2_ to OH radicals (Figure 1e), they have been shown to activate H_2_O_2_ via a nonradical pathway, forming surface peroxo species. The absence of TMB oxidation for CeO_2_ octa. (Figure 1f) further suggests that these peroxo species cannot oxidize TMB due to its high redox potential (+1.130 V_NHE_).^49^ However, they were found to selectively oxidizes Br^–^ (+0.76 V_NHE_)^50^ to HOBr, mimicking bromoperoxidase (BPO), with reactivity correlated to the electron deficiency (or Lewis acidity) of the active sites (Figure 1c­(**ii**)). Since the oxidation of Br^–^ by OH radicals does not produce HOBr,^51^ the BPO-like activity of CeO_2_ shapes is reversed, with the octa outperforming the sphere, while the cube shows negligible activity (Figure 1i).

These findings indicate that H_2_O_2_ activation occurs exclusively via the radical pathway in the cube and the peroxo pathway in the octa (Table 1). Such opposing behavior in H_2_O_2_ activation is attributed to their uniform yet electronically distinct Ce sites, as evidenced by the distinct, nonoverlapping nature of their ^31^P NMR signals (Figure 1h).^41^ This is critically important for mechanistic studies, as nanozymes without precise shape control may have active sites with varying reactivity, hindering reliable catalytic correlation. For example, the broad ^31^P signal of the sphere, centered between the cube and octa, suggests the presence of various Ce sites—some activating H_2_O_2_ via the radical pathway and others via the peroxo pathway. Since not all Ce sites can effectively contribute to either pathway, this sample, despite having the highest surface area (Figure 1d), exhibits dual but poor POD-like (Figure 1f) and BPO-like (Figure 1i) activities.^39^ This example also clarifies why some reports mistakenly correlated OH radicals with HOBr yield,^52,53^ even though HOBr can be produced only via the peroxo pathway. This incorrect correlation was attributed to nonuniform Ce sites with varying electron richness in their nanozymes, as well as the absence of control experiments. Similar regulation of Ce sites, and hence precise control of H_2_O_2_ activation, has also been recently demonstrated by Cheng et al.^54,55^

### Catalytic Correlation for Cluster and Single-Atom Nanozymes

The electronic regulation of active sites, and consequently the activation pathway of H_2_O_2_, can be further extended to explain the differences in POD-like activity when a given metal “M” is present as a cluster versus its single-atom form in nanozymes. While single-atom nanozymes have gained attention for maximizing the utilization of active sites and enhancing our understanding of reaction mechanisms, much of their POD-like activity is attributed to the production of OH radicals, akin to that of conventional POD nanozymes,^56–61^ even for those designed to mimic the heme center of HRP. In fact, these single-atom centers, often coordinated by electronegative O/N/C atoms, carry a positive charge and exhibit lower electron density than their metallic counterparts in cluster form. This impedes the reduction of H_2_O_2_ to OH radicals, ultimately leading to lower POD-like activity. For example, we found that Ru single atom coordinated by three oxygen atoms (denoted as Ru–O_3_) exhibit positive charges ranging from +1.12 to +1.36|*e*| (Figure 2a, left).^62^ In contrast, the top Ru atom of Ru_4_ cluster, which does not have direct contact of oxide support, exhibits metallic character, with a Bader charge close to 0|*e*| (Figure 2a, right). Although POD-like activity generally increases with overall Ru concentration in the samples (from A to D, Figure 2b), the normalized activity (or Ru atom efficiency) peaks in samples where metallic Ru is present as clusters (i.e., samples B and C). Increasing the size to nanoparticles in sample D reduces Ru atom efficiency due to fewer exposed metallic Ru atoms available for the reaction. Surprisingly, the Ru single-atom configuration in sample A exhibits the lowest Ru atom efficiency despite all Ru sites participating in the reaction. This was attributed to the positively charged Ru, which slows H_2_O_2_ reduction and hinders the production of OH radicals via radical pathway for TMB oxidation (Table 1). Despite this, the surface peroxo/oxo species formed via nonradical pathways may oxidize other substrates with lower redox potentials, as demonstrated by the CeO_2_ octa above.

**Figure 2:**
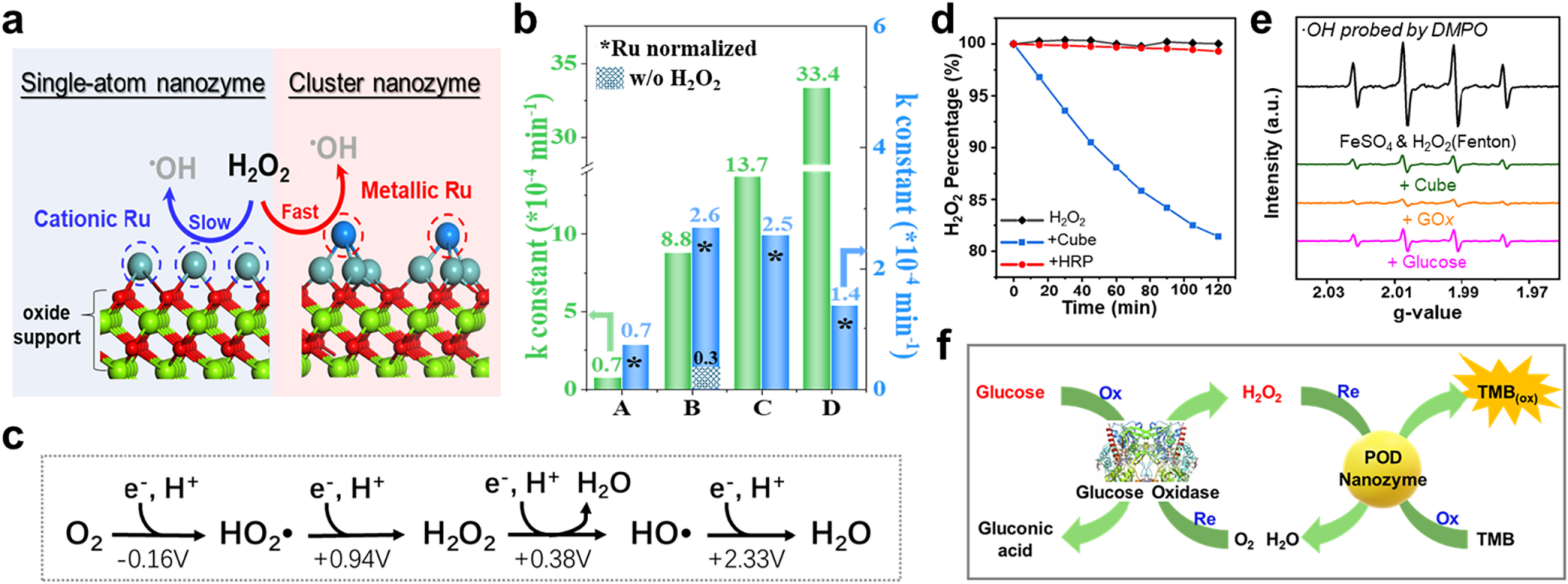
(a) Schematic illustration of the conversion of H_2_O_2_ to OH radicals over Ru-based single-atom and cluster nanozymes. (b) Comparison of the rate constant *k* before (green column) and after (blue column) normalizing Ru concentrations. Figures (a, b) reproduced with permission from ref 62. Copyright 2022 Wiley-VCH. (c) Sequential reduction of O_2_ and the corresponding redox potentials. (d) Decomposition of H_2_O_2_ in the presence of CeO_2_ cube and HRP. (e) OH radical scavenging in the presence of CeO_2_ cube, GO*x*, and glucose. (f) The working principle of the colorimetric assay for glucose detection.

In addition to exhibiting POD-like activity, metallic Ru in cluster form was also found to reduce O_2_, a process that is more challenging than the reduction of H_2_O_2_ (Figure 2c),^63^ thereby demonstrating additional oxidase-like activity. For example, in sample B, while O_2_ reduction competes with H_2_O_2_ reduction, the impact on its POD-like activity is minimal, as evidenced by an oxidase-like activity of only 0.3 units (see Ru-normalized absorbance, Figure 2b), which represents just 11.5% of 2.6 units observed from its POD-like activity. This is attributed to the lower concentration and weaker adsorption of dissolved O_2_ versus the high concentration (100 mM) and strong adsorption of H_2_O_2_.^62^ Notably, even with this competition, the POD-like activity of the Ru cluster sample (sample B) remains markedly higher than that of the Ru single-atom sample (sample A). Since reducing H_2_O_2_ is much easier than reducing O_2_, some studies have thus claimed that their single-atom nanozymes can achieve high specificity for POD-like activity with suppressed oxidase-like activity, as observed in our Ru single-atom sample. For example, Wang et al. reported that a molybdenum center coordinated by three nitrogen atoms and one carbon atom (Mo–N_3_-C) exhibits selective POD-like activity without oxidase-like function.^64^ Similar control was also reported by Lee’s group employing an iron center coordinated by four nitrogen atoms (Fe–N_4_).^65^ Both results suggest that their single-atom centers are not electron-rich enough to reduce O_2_ but H_2_O_2_, thereby specifically exhibit POD-like activity. Although turnover frequency (TOF) was not reported for these single-atom nanozymes, the positive charge of their active site is expected to impede radical H_2_O_2_ activation, resulting in poor POD-like activity. Interestingly, a recent study showed that Fe–N_4_ sites positioned at the edges of single-atom nanozymes can exhibit both POD- and oxidase-like activities.^66^ Given that these edge Fe–N_4_ sites are sufficiently electron-rich to reduce O_2_, the POD-like activity of this sample, in terms of TOF, should surpass that of the two previous single-atom nanozymes (Table 1). These findings, along with the fact that a material capable of reducing O_2_ should also reduce H_2_O_2_ (Figure 2c), explain why oxidase-mimicking nanozymes often exhibit POD-like activity, whereas POD nanozymes may not show oxidase-like activity.^3–6^

## Limitations

Although Fenton-based POD nanozymes with greater electron richness at their active sites are expected to display better activity, they remain intrinsically limited by the disadvantage of the Fenton reaction, including low H_2_O_2_ utilization and poor anti-interference capabilities associated with OH radicals (Scheme 2). Unlike HRP, which uses H_2_O_2_ for stoichiometric TMB oxidation, recent literature argues that the addition of TMB (or other dyes) serves only a colorimetric reporter for evaluating the rates of H_2_O_2_ decomposition in these materials.^67,68^ This statement can be supported herein using CeO_2_ cubes, which exhibit the highest POD-like activity among CeO_2_ shapes (Figure 1). As shown in Figure 2d, the sample indeed decomposes H_2_O_2_ over time in the absence of TMB, whereas HRP shows no change in H_2_O_2_ concentration without TMB. In addition to this issue, OH radicals are known to be scavenged by not only themselves but also various substances (Scheme 2). As shown in Table 2, OH radicals can recombine to regenerate H_2_O_2_ (entry 1) or undergo self-redox reactions, yielding O_2_ and H_2_O (entry 2). They may even be removed by the Fenton catalyst itself (e.g., “M^n+^” in entry 3). To support this, we generated OH radicals using a typical Fenton system (Fe^2+^ + H_2_O_2_) and tracked their quantity through electron paramagnetic resonance (EPR) using DMPO (5,5-dimethyl-1-pyrroline-N-oxide).^69–71^ As expected, the EPR signals of OH radicals significantly decrease in the presence of CeO_2_ cube (Figure 2e), likely scavenged by the surface electron-rich Ce sites. Altogether, in a simple solution system comprising H_2_O_2_, TMB, and POD nanozymes, OH radicals can be scavenged through various reactions beyond just TMB considered in the literature.^3–11^ A recent study further calculated that in a solution with 10 mM H_2_O_2_ and 1 mM TMB, Fe_3_O_4_ nanozymes decomposed 1.97 mM H_2_O_2_ in 20 min, yielding only 0.089 mM of TMB_ox_ (ca. 4.5% of the consumed H_2_O_2_), significantly lower than the 17% expected based on its proportion in the stock solution (i.e., [TMB]/([TMB]+[H_2_O_2_])).^72^ This stands in stark contrast to HRP, which stoichiometrically utilize H_2_O_2_ for TMB oxidation in a one-to-one manner.

**Scheme 2:**
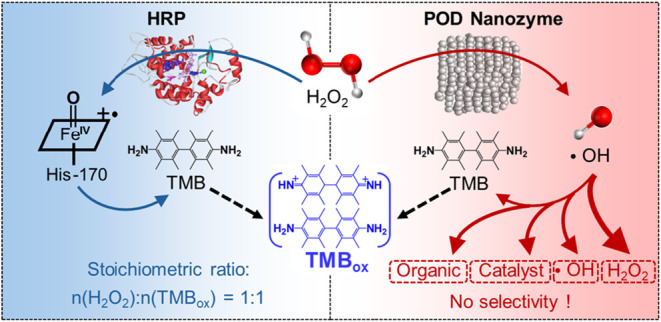
Schematic Illustration of the Difference in Mechanism between HRP and Fenton-Based Nanozymes in TMB Oxidation with H_2_O_2_

**Table 2:** Processes Scavenging OH Radicals^27^

OH radical scavenging processes not included in Scheme 1f		
between radicals	${\mathrm{HO}}^{•}+{\mathrm{HO}}^{•}\to H_{2}O_{2}$	entry 1
	$2{\mathrm{HO}}^{•}+2{\mathrm{HO}}^{•}\to O_{2}+2H_{2}O$	entry 2
removal by substances	$M^{n+}\left(c\mathrm{atalyst}\right)+{\mathrm{HO}}^{•}+H^{+}\to M^{\left(n+1\right)+}+H_{2}O$	entry 3
	$o\mathrm{rganic}+{\mathrm{HO}}^{•}\to {\mathrm{organic}}_{\mathrm{ox}}+H_{2}O$	entry 4

In the literature, POD nanozymes are often coupled with various oxidase enzymes for colorimetric sensing of biologically important molecules.^9^ For example, in glucose detection (Figure 2f), glucose is first oxidized by glucose oxidase (GO*x*), resulting in the stoichiometric production of H_2_O_2_ from O_2_. In the second step, POD nanozymes utilize this H_2_O_2_ to oxidize TMB, leading to a color change. Since the degree of color change is believed to reflect glucose concentration, researchers have thus derived the sensing parameters (e.g., sensitivity and limit of detection) of their POD nanozymes for performance comparison. However, as discussed above, POD nanozymes cannot stoichiometrically utilize the H_2_O_2_ produced by GO*x* for TMB oxidation as that of HRP, which can lead to the underestimation of actual glucose concentrations. Moreover, organic matters added in the detection system may also react with OH radicals (see Table 2, entry 4). As also demonstrated in Figure 2e, OH radicals produced by typical Fenton system (Fe^2+^ + H_2_O_2_) were greatly removed by GO*x* and glucose, as evidenced by the sharp decrease in EPR signals probed by DMPO following their addition. The removal of OH radicals by these substances would further reduce H_2_O_2_ utilization for TMB oxidation, which inherently limits the sensitivity of these Fenton-based POD nanozymes, especially at low substrate/H_2_O_2_ concentrations. In practice, the sensitivity of Fenton-based POD nanozymes could thus be severely constrained by the complexity of real biological fluids and food matrices. These environments contain a wide range of OH radical scavengers—such as amino acids (e.g., histidine, cysteine), reducing sugars, natural antioxidants (polyphenols, glutathione), and proteins—making the radicals’ half-lives as short as femto-picoseconds.^73–77^ This intrinsically hinders the replacement of HRP with these Fenton-based POD nanozymes in practical assays.

## Conclusions and Outlook

The design and synthesis of solid Fenton catalysts that efficiently convert H_2_O_2_ into OH radicals have been well-documented over the past decades. As interest grows in substituting HRP with POD nanozymes in related sensing applications, their Fenton-based mechanism suggests that their design for better activity should adhere to the same principles. Consequently, strategies previously demonstrated to be effective for Fenton catalysts^78^ are believed to also improve the POD-like activity of nanozymes (i.e., a good Fenton catalyst would be a good POD nanozyme). Regarding surface characterization, we encourage researchers in the field to adopt probe-assisted techniques, alongside XPS, as complementary tools often used in heterogeneous catalysis to gain deeper insights into the catalysts’ surface chemical states. The incorporation of situ/operando techniques, such as EPR and X-ray absorption spectroscopy (XAS), could further help establish more robust catalytic correlations. In the second part, we highlighted the challenges associated with current Fenton-based POD nanozymes, particularly regarding their H_2_O_2_ utilization and potential interferences from various species, both of which intrinsically render them less than ideal as HRP mimetics. The Michaelis–Menten analysis may thus be mathematically inappropriate for them, as the steady-state assumptions governing substrate consumption are not directly tied to catalyst turnover. While confined catalysis and other strategies have proven effective for improving H_2_O_2_ utilization of Fenton-based catalysts in AOP pollutant removal,^79^ analytical sensing aims more strictly to stoichiometrically utilize H_2_O_2_, a goal inherently limited by radical-mediated activation. We therefore argue that Fenton-based POD nanozymes are fundamentally unsuitable as HRP substitutes, especially in quantitative assays.

To address this, nonradical POD nanozymes that activate H_2_O_2_ via a peroxo/oxo-mediated pathway have recently gained attention. In addition to the CeO_2_ cases discussed above, spin regulation of atomically dispersed Fe­(II) species on nitrogen-rich graphene has been reported to modulate adsorption affinity toward H_2_O_2_, facilitating its activation via a peroxo pathway while suppressing the radical pathway.^80^ Li et al. recently demonstrated that incorporating Fe into TiO_2_ generates oxygen vacancies, which facilitate the formation of reactive Ti-peroxo species, thereby enabling TMB oxidation via a nonradical pathway.^81^ For pristine TiO_2_, light irradiation serves as another approach to generate Ti-peroxo species that are sufficiently reactive (enabled by photogenerated holes) to oxidize TMB.^82^ The entire catalytic cycle can be further promoted by scavenging photogenerated electrons with Ag^+^, a strategy that was utilized for Ag^+^ determination.^83^ Although the weak oxidizing power of peroxo/oxo species may limit substrate scope and reaction rates, they are expected to utilize H_2_O_2_ more efficiently in a manner similar to HRP (Scheme 2), thereby improving selectivity and quantitative accuracy. Additionally, while the high redox potential of TMB (+1.130 V_NHE_) may exceed the oxidizing capacity of most surface peroxo/oxo species, alternative colorimetric substrates with lower redox potentials can be used, such as 2,2′-azino-bis­(3-ethylbenzothiazoline-6-sulfonic acid) (ABTS, + 0.68 V_NHE_)^84^ and o-phenylenediamine (OPD, + 0.337 V_NHE_).^85^ The feasibility of this approach was previously demonstrated by our group, showing that although the surface peroxo species of CeO_2_ octa cannot oxidize TMB (Figure 1f), they selectively oxidize OPD without decomposing H_2_O_2_.^86^ Similarly, although positively charged metal centers (e.g., Ru–O_3_, Mo–N_3_-C, and Fe–N_4_ sites) inherently disfavor the radical pathway for H_2_O_2_ activation on single-atom nanozymes, the resulting surface peroxo/oxo species may selectively oxidize ABTS or OPD, thus improving H_2_O_2_ utilization and sensing accuracy in the coupled reaction (Figure 2f). Overall, nonradical POD nanozymes are expected to outperform their Fenton-based counterparts, particularly for quantitative purposes, by shifting H_2_O_2_ activation from maximizing radical production to enhancing the reactivity of surface peroxo/oxo species, which requires an alternative design strategy.

In fact, heterogeneous catalysis realm has established a wealth of knowledge on designing solid catalysts that regulate H_2_O_2_ activation to improve its utilization and substrate selectivity in oxidation reactions.^87–89^ For example, redox-inactive oxides such as Ti, Zn, Zr, and Nb are known to inherently bypass the radical pathway, enabling selective oxidization of target substrates with high H_2_O_2_ utilization (Figure 3a). The reactivity of the resulting peroxo species toward substrates can be further tuned by regulating surface features such as adsorbates and exposed facets. Using cyclohexene epoxidation as a probe reaction, Sun et al. recently showed that surface peroxo species on anatase TiO_2_ exhibit various configurations with distinct reactivities (Figure 3b):^87^ the activity on fluoride-modified (001) surfaces is significantly higher than on pristine (001) surfaces, while the (101) surface shows negligible oxidation activity. Additionally, bulk-to-nano regulation of layered HNb_3_O_8_ materials has been reported to distort surface NbO_*x*_ units, thereby exposing Nb sites with Lewis acidity proportional to the degree of structural distortion (Figure 3c).^88^ Among the various surface Nb-peroxo species, the bidentate configuration formed on highly distorted NbO_*x*_ of s-HNb_3_O_8_ demonstrated the highest reactivity for cyclohexene epoxidation, achieving near-stoichiometric H_2_O_2_ utilization. Additionally, based on Scheme 1e, the generation of the highly reactive Fe­(IV)=O radical species (compound (ii)) is closely related to both the iron active site and the amino acids precisely arranged in the secondary coordination sphere. Mimicking the HRP microenvironment—such as embedding Fe-porphyrin in a tailored organic ligand framework or engineering metal–organic frameworks (MOFs)—could serve as an alternative approach. Applying these strategies in the design and synthesis of nonradical POD nanozymes would better mimic HRP’s catalytic behavior, thereby optimizing their H_2_O_2_-to-substrate stoichiometry and accuracy in sensing applications.

**Figure 3:**
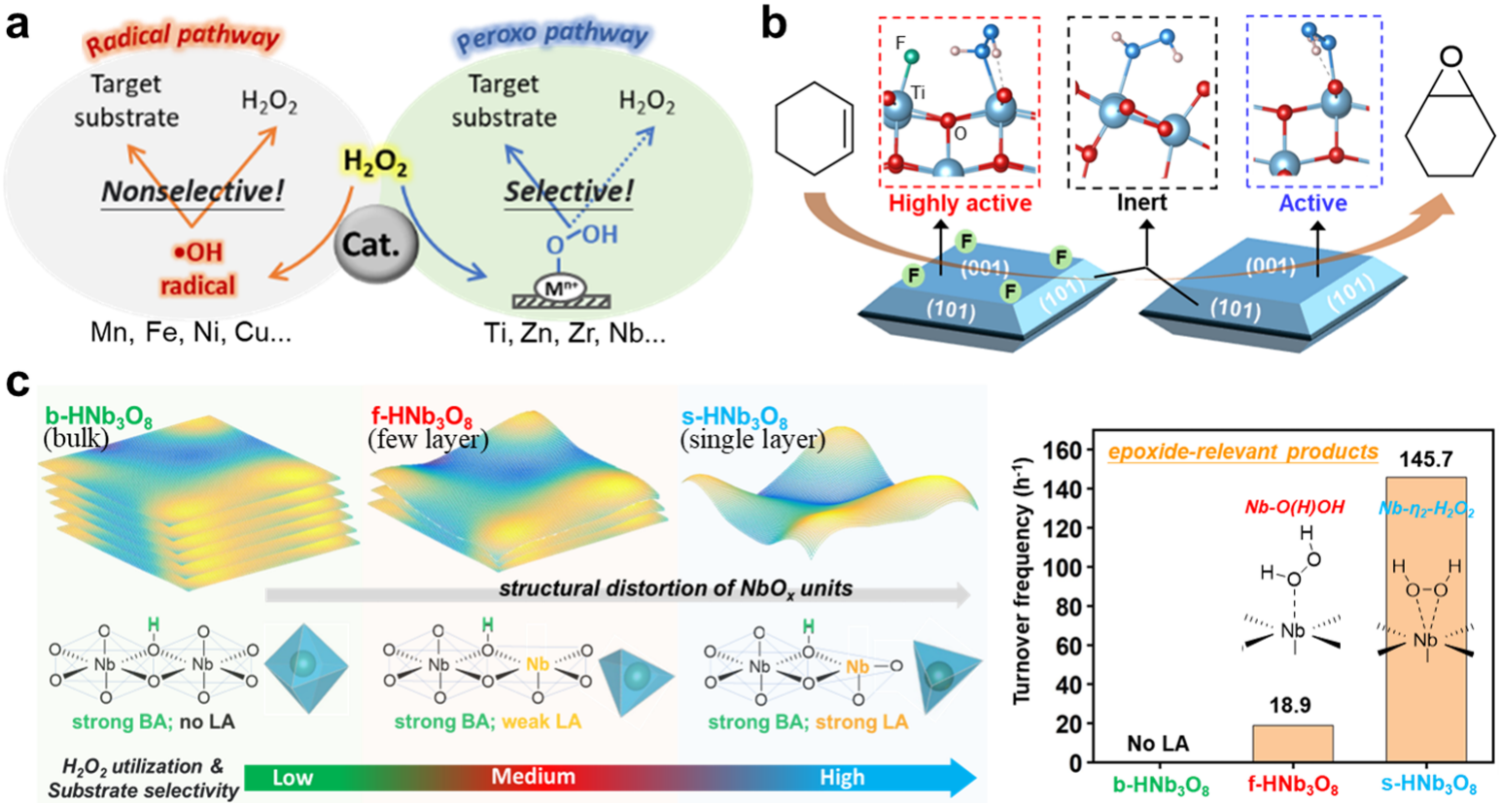
(a) H_2_O_2_ activation pathways over redox-active versus redox-inactive metals. (b) Formation of surface peroxo species with different configurations over anatase TiO_2_ and their reactivity in cyclohexene epoxidation. Reproduced with permission from ref 87. Copyright 2024 American Chemical Society. (c) Bulk-to-nano regulation of layered HNb_3_O_4_ and the corresponding activity for cyclohexene epoxidation. Reproduced with permission from ref 88. Copyright 2022 Elsevier.
